# 5q Minus Myelodysplasia Associated with Multiple Epithelioid Granulomas within Conventional Renal Cell Carcinoma

**DOI:** 10.1155/2012/138126

**Published:** 2012-07-16

**Authors:** Rahul G. Matnani, Roshan K. Patel, Stephen E. Strup, Rouzan G. Karabakhtsian

**Affiliations:** ^1^Department of Pathology and Laboratory Medicine, College of Medicine, Chandler Medical Center, University of Kentucky, 800 Rose Street, Lexington, KY 40536-0298, USA; ^2^Division of Urology, Department of Surgery, College of Medicine, Chandler Medical Center, University of Kentucky, 800 Rose Street, Lexington, KY 40536-0293, USA

## Abstract

A 69-year-old Caucasian female, with a previous diagnosis of 5q minus myelodysplastic syndrome, presented with conventional renal cell carcinoma (RCC) associated with multiple-epithelioid nonnecrotizing granulomas. Two previous reports of sarcoidosis, primarily involving the lung and skin, have been described in patients with 5q minus myelodysplasia. A cluster of closely linked genes encoding for cytokines such as IL-4, IL-5, and IL-3 are present on chromosome 5q. Hence, in sarcoidosis, cytokine imbalances associated with the deletion of these cytokine genes have been postulated. However, an occurrence of epithelioid granulomas within a carcinoma, in preexisting clonal myelodysplastic syndrome, has not been described. The patient, in the current study, had long standing 5q minus deletion, clinically characterized by refractory anemia associated with hypolobated megakaryocytes. However, the patient's history was negative for sarcoidosis and the extensive nonnecrotizing epithelioid granulomas were confined within RCC. Due to the absence of Th-2 cytokines, such as IL-4 and IL-5, in a subset of 5q minus myelodysplastic syndrome, proinflammatory Th-1 cytokines such as IFN-**γ** and TNF-**α** may be exaggerated in an environment conducive to antigen expression. Hence, we propose a greater susceptibility for the development of granulomas, at least in a subset of patients with 5q minus myelodysplasia.

## 1. Introduction

Ineffective hematopoiesis in myelodysplasia is characterized by hypercellular bone marrow and peripheral blood pancytopenia. The 5q deletion in myelodysplasia, in the absence of other complex cytogenetic abnormalities, is associated with a favorable prognosis or with a low risk for development of leukemia [1, 2]. Two previous reports linking 5q minus syndrome and sarcoidosis have been described [3, 4]. In both reports, sarcoidosis involved the lung and skin. However, three patients with a known history of nonclonal myelodysplasia have later developed disseminated granulomatous skin eruptions [5, 6]. Interestingly, multiple-gene encoding for cytokines such as IL-4, IL-5, and IL-3 and receptors for different growth factors such as platelet derived growth factor (PDGF) are closely linked on long arm of chromosome 5 [7, 8]. The dominant role of proinflammatory Th-1 cytokines such as IFN-*γ* and TNF*α* has been well documented in the pathogenesis of granuloma formation in various animal and human studies [9–12]. Hence, in absence of Th-2 cytokines (IL-4 and IL-5), cytokine imbalances associated with exaggerated Th-1 cytokines may occur in a subset of 5q minus myelodysplastic syndrome. This coupled with an environment favoring antigen expression, may lead to sustained macrophage and T-cell activation and subsequent granuloma formation. We report a case of multiple granulomas associated with RCC in a patient with 5q minus myelodysplasia.

## 2. Case Report

The patient is a 69-year-old lady with a previous diagnosis of clonal myelodysplasia, specific for 5q deletion, in the setting of refractory normocytic anemia. The bone marrow biopsy performed in 2007 showed small hypolobated megakaryocytes with bone marrow chromosomal analysis and fluorescence in situ hybridization (FISH) demonstrating deletion of 5q. The patient, however, was not treated with lenalidomide for 5q minus myelodysplastic syndrome due to previous history of pulmonary embolism and stroke. In addition, her past history was significant for hypertension, diabetes, and a history of brain aneurysm repair in 1995. The patient also reported allergy to sulfa drugs. The patient stopped smoking in 2002 and denied any alcohol use. The family history was negative for leukemia or myelodysplastic syndrome. In October 2011, she underwent a CT scan for left flank pain which revealed a left lower pole mass suspicious for RCC. At the time of surgery, in December 2011, the patient had refractory normocytic anemia with hemoglobin of 8.7 g/dL. However, all other hematological, biochemical, and serological parameters were normal. The patient did not have any history of sarcoidosis, tuberculosis, or history of immunization with Bacillus Calmette-Guérin (BCG). Clinical symptoms, additional imaging, and laboratory findings were negative for sarcoidosis.

## 3. Pathologic Findings

**Figure 1:**
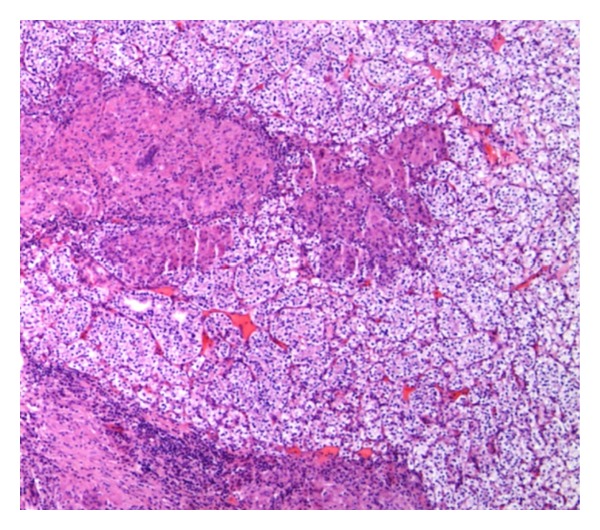
Conventional renal cell carcinoma of clear cell type with Fuhrman grade II nuclei and associated multiple granulomas (40x magnification).

**Figure 2:**
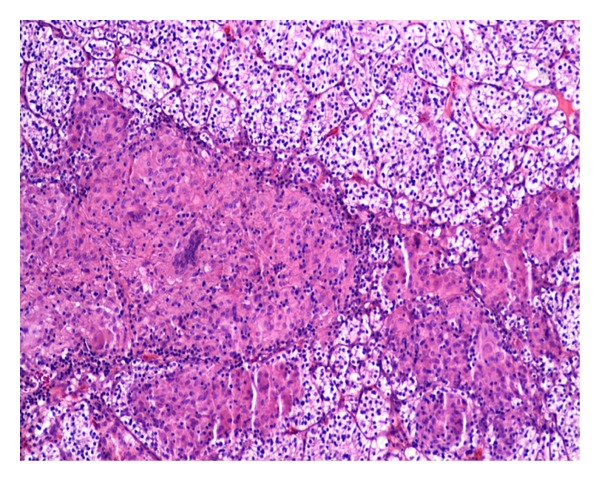
Variable-sized multiple epithelioid nonnecrotizing granulomas within conventional renal cell carcinoma (100x magnification).

**Figure 3:**
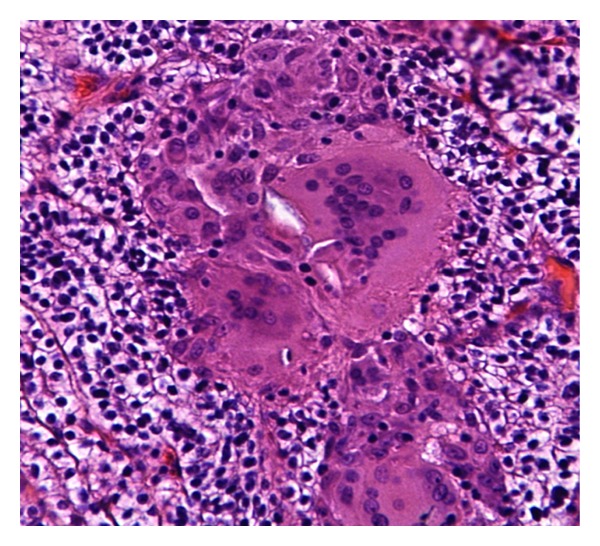
Epithelioid nonnecrotizing granulomas with multinucleated foreign body type giant cells (200x magnification).

On gross examination, a fairly well-circumscribed tumor measuring 6.0 × 5.3 × 4.5 cm was present at the lower pole of the left kidney. The cut surface appeared yellow-red with a central area of hemorrhage. The tumor was confined to the kidney. The remaining uninvolved kidney was grossly unremarkable. Microscopically, the tumor showed features of conventional RCC of clear cell type with Fuhrman grade II nuclei (Figure 1). There was no evidence of lymphvascular invasion. Interestingly, there were multiple discrete epithelioid nonnecrotizing granulomas of variable size within the tumor (Figure 2). The granulomas demonstrated numerous multinucleated giant cells of foreign body type with lymphocytic infiltration (Figure 3). There was no evidence of asteroid or Schaumann bodies within the granulomas. The Gomori-Grocott methenamine silver stain and Fite's acid fast stain did not reveal any fungal or mycobacterial organisms. The normal kidney parenchyma did not show any evidence of granulomas. 

## 4. Discussion

Noncaseating granulomas in association with breast, liver, and colon carcinomas have been described [13–15]. Non-necrotizing granulomatous reaction within the renal cell carcinomas (RCC) is rare with only a few published reports in the literature [16–20]. The RCC described by Marinides et al. was of a papillary type. There is one case report of nonnecrotizing granulomas in RCC with sarcomatoid features [21]. However, RCC in other reports, similar to our case, had a clear cell pattern with the presence of epithelioid granulomas. The current case report may indicate a possible association between preexisting clonal myelodysplasia and granulomatous reaction within the RCC.

Our patient has a long-standing history of clonal (5q minus) myelodysplastic syndrome associated with refractory normocytic anemia and small hypolobated megakaryocytes. The granulomas were exclusively associated within the RCC tumor and were entirely absent in the surrounding nonneoplastic renal parenchyma. Previous case reports of granulomatous RCC in a preexisting myelodysplasia have not been reported. However, a few case reports of sarcoidosis involving lung and skin have been associated with 5q minus syndrome [3, 4]. In our case, the patient did not have any history or symptoms suggestive of sarcoidosis. Except for a long standing refractory anemia, the biochemical, serological, and hematological parameters were within normal limits. The cases reported by Tunkel et al. and Airaghi et al. along with our present case report suggest a possible association between epithelioid granulomas and 5q minus myelodysplastic syndrome. The possibility of this association is further strengthened based on the evidence of clustering of genes encoding for Th-2 cytokines on long arm of chromosome 5. Hence, deletion of these genes may lead to cytokine imbalance and initiation of granuloma formation upon antigenic stimulation. Moreover, nonclonal myelodysplasia has been shown to be rarely associated with disseminated nonnecrotizing granulomatous skin eruptions [5, 6]. Hence, propensity to develop noncaseating epithelioid granulomas, in the setting of myelodysplasia, warrants further investigation. 

In conclusion, this is the first reported case of multiple granulomas confined to a renal cell carcinoma in a patient with preexisting myelodysplastic syndrome. The hypothesis of existence of cytokine imbalance and uncontrolled activation of T cells and macrophages, in the setting of 5q minus myelodysplasia has been previously proposed [3]. The present study provides further support to this hypothesis. However, unlike previous studies, these granulomas were confined to the renal cell carcinoma in the setting of preexisting blood dyscrasia. Further studies may potentially look more closely at the possible association of myelodysplasia and immune activation leading to formation of epithelioid granulomas.
